# Gregory O’Brien MB ChB MA MD FRCPsych FRCPCH FRANZCP

**DOI:** 10.1192/pb.bp.114.050054

**Published:** 2015-06

**Authors:** Tom Berney

**Figure:**
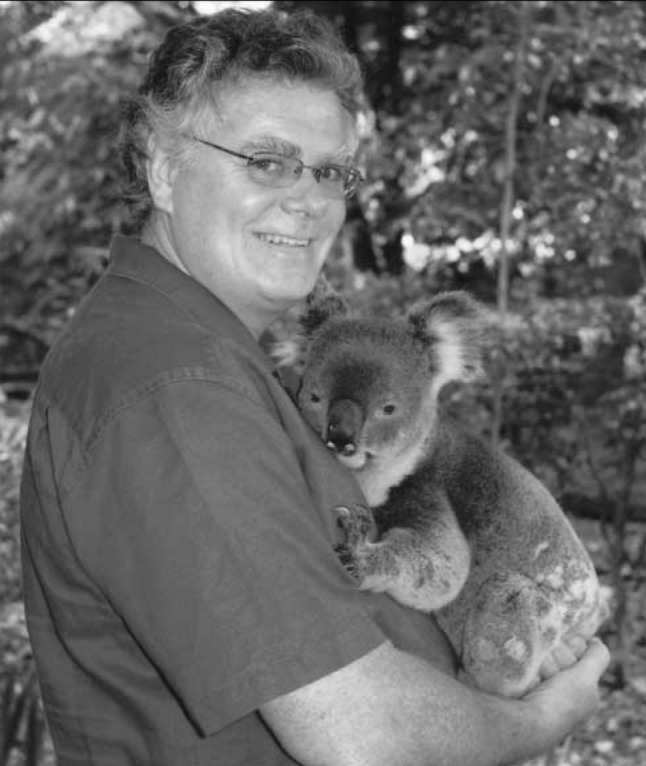


Formerly Professor of Developmental Psychiatry, Northumbria University and the University of Queensland; Consultant Psychiatrist and Associate Medical Director, Northumberland, Tyne and Wear NHS Foundation Trust; Senior Psychiatrist with the Queensland Mental Health Assessment and Outreach Team of Disability Services.

Greg O’Brien, who died recently aged 59 years, played a major part in developing the character of the speciality of the psychiatry of intellectual disability, first in the UK and then in Australia.

Greg entered intellectual disability psychiatry in the 1980s at a time when its role and future were being questioned. Over the next 30 years, he helped develop its clinical and academic character. Within the Royal College of Psychiatrists, he was elected Chair of the Faculty of Psychiatry of Intellectual Disability in 2002 and appointed Associate Dean in 2006, an office that gave him a wider opportunity to develop psychiatric education. He was appointed to the academic chair in Developmental Psychiatry at Northumbria University in 2004. Martin Bax drew him into the Society for the Study of Behavioural Phenotypes and the Ronnie MacKeith group where he was able to focus on autism and epilepsy. These various worlds had in common a clannish, crusading nature, which Greg explored with energy and ebullience, fermenting ideas through encounters that included long journeys, long lunches, research meetings and conferences. His numerous publications reflect a wide range of concepts and approaches to clinical practice. In particular, he was the senior editor of *Behavioural Phenotypes*, the first textbook published on this subject.

The College gave Greg a further forum which he relished, enjoying its influence on psychiatric training and service development. Like others, he had set out to be a child psychiatrist, only to be drawn into the fast-changing, multidisciplinary adventure of intellectual disability. Both locally as a medical director and nationally as the faculty chair, he was effective in steering professional and service development through the surf of repetitive reconfiguration that characterises the NHS. His apparent confidence in doing this was achieved only by careful preparation.

An unfashionable specialty, Greg made sure the Faculty of Psychiatry of Intellectual Disability would be a significant source of support for many isolated members. Its growth inevitably brought some loss of informality and intimacy. Greg’s antidote was to foster the inclusive, all-are-welcome ethos that was the Faculty’s hallmark. He started the tradition of after-dinner music at Faculty meetings, the sessions extending through the night and becoming, for some, the highlight of the programme.

While still at the height of this career, he left the UK in 2010 to move to Queensland, where he succeeded in recapturing the pioneering spirit that had inspired him in the UK, developing a new service and its academic base. Appointed Senior Staff Specialist on the Mental Assessment and Outreach Team of Disability Services and to the chair in Developmental Psychiatry at Griffith University, he set about a similar campaign to convince the Royal Australian and New Zealand College of Psychiatrists not only that there were powerful intellectual challenges in neurodevelopmental psychiatry but also that this could become the most human and clubbable of the psychiatric specialties.

Greg grew up in Paisley, where his family and school (St Aloysius College) left him with the strong sense of social justice that would underpin his life. His father, Jack O’Brien, was a Glasgow trade unionist. After graduating from Aberdeen Medical School, Greg entered psychiatry in Newcastle. There he met Barbara, who was to become a specialist in paediatric intensive care, and whom he later married. The O’Briens moved to Cambridge in 1986, where Greg took up a post as lecturer in the Department of Psychiatry, specialising in intellectual disability. In 1991, he returned to Newcastle to become a consultant at Northgate Hospital. There, with Ken Day as his mentor, he focused on forensic psychiatry and medical management. At the same time, he completed his MD and set about fostering academic development in his specialty, directing the regional training programme, organising research, editing a series of books and managing the politics of a professorial chair.

Throughout his career, Greg remained a clinician, seeing, treating and learning from individuals, their families, friends and carers. He had an intense enthusiasm combined with the strong sense of humour that carried him through his career. Behind the jollity and extravagance of manner was a keen awareness that he was lucky to have such congenial employment. He was always ready to help colleagues with advice that was not only intelligible but also constructive. He was a great confidant, recognising the normality of imperfection. For Greg, however, the real world was rooted in his family and friends whose own lives were shaped by his warmth and humanity.

His illustrated life on Facebook, written from Australia, was about fun, friends and family (blithely ignoring the cancer that developed a year after his arrival) and he lived life to the full, right up to the point when he wrote a dignified letter of farewell to the Australian College (https://www.ranzcp.org/Membership/Subspecialty-groups/Interest-Groups/Intellectual-Developmental-Disabilities/SIGPIDD-Newsletter-May-2014.aspx). He returned to Newcastle and made arrangements for his disposal (a funeral mass at the cathedral, a crowded wake and then, on the following day, a more private cremation).

Greg O’Brien died of cancer on 13 July 2014 at his home in Newcastle upon Tyne. He is survived by his wife, Barbara, and their children, Áine and Daniel.

